# Pricing the national health insurance scheme in Qatar – opportunities and challenges

**DOI:** 10.1186/1472-6963-15-S2-A6

**Published:** 2015-06-15

**Authors:** Finn Goldner, Jim Pearse, Deniza Mazevska

**Affiliations:** 1 National Health Insurance Company, Doha, Qatar; 2 Health Policy Analysis, Sydney, 2065, Australia

## Background

In 2013, Qatar introduced a national health insurance scheme, called “Seha.” Stage 1 of the scheme covered women aged 12 years and older for health care and maternity services. Stage 2 was launched in 2014, and covered all Qatari nationals for a much broader set of services. In the future, Stages 3 and 4 will extend coverage to all non-Qatari residents within the country as well as visitors.

The National Health Insurance Company (NHIC) manages Seha, with a Third-Party Administrator (TPA) contracted to manage provider claims. The Supreme Council of Health (SCH) acts as the scheme’s overall regulator, and is responsible for activities such as specifying coverage and approving pricing.

A range of steps was taken to prepare for Seha’s launch. SCH mandated clinical coding using ICD-10-AM, and hospitals recruited and/or trained clinical coders to use this system. The Australian Refined Diagnosis Related Groups (AR-DRGs) tool was adopted, and public sector hospitals embarked on the clinical costing of their services.

In Stage 1, SCH decided to use a bundled payment method for health care services. AR-DRGs were chosen for pricing acute inpatient care, using 76 AR-DRGs relevant for women and maternity services. A modification of the Australian Tier 2 classification was adopted for specialist medical services, using 9 classes relevant to women’s health. In addition, a primary health classification was adopted based on 4 levels of complexity. Mammography and MRI were unbundled from the specialist and primary care service, but other imaging, laboratory, and pharmacy services were bundled into the price. Although this was not included in Stage 1, previous work had also recommended the adoption of Urgency Related Groups (URGs) classification for emergency care.

Stage 1 was implemented with a limited network including both private and public hospitals. The launch of Seha involved providing information to prospective providers and conducting subsequent sessions to assist in operationalizing the price schedule and business rules.

Seha’s challenges for Stage 2 included: pricing a much wider range of services than were included in Stage 1; expanding the scheme to a wider range of providers, including “stand alone” providers (i.e. those without the capacity to provide ancillary services); and addressing the limited availability of activity and cost information from the private sector.

## Materials and methods

This paper outlines the approach to pricing Stage 2, including some of the challenges encountered, and discusses the next steps for Seha’s pricing over the next financial year and beyond.

An initial step involved consultations with both public and private providers. A benchmarking study of international prices for comparable services was also undertaken to provide a basis for understanding the extent to which prices and relativities aligned with other countries both within the region and around the world.

The authors obtained available activity information from the major government providers. AR-DRG cost estimates were available from a previous study undertaken at the major government hospitals (although these were impacted by the fact they were not based on ICD-10-AM coded data). High-level cost estimates, which were available for other care streams, were used towards pricing.

Similar data from private providers were not always available. Instead, researchers sought to use current price lists, aggregate costs, and summary data (where unit record activity data were not available) from these providers.

## Results

Various payment policy options were explored and advice developed. These included issues such as unbundling pharmacy costs from specialist and general practice costs and creating differential pricing for initial, subsequent, and repeat specialist attendances. In addition, a price schedule was developed for the full range of admitted and non-admitted services, accompanied by a comprehensive set of business rules.

The information being gathered in Stage 2 will be used as the basis for pricing services and fine-tuning business rules to improve the existing methodology and prepare for Seha’s next stage. Using the standard network agreement, NHIC has gained a commitment from all providers to report their costing data to SCH, which will provide additional information towards pricing.

## Conclusions

The design of the payment system underpinning Seha was ambitious in beginning with a bundled model across both inpatient and outpatient sectors. The desire for a bundled system had to be balanced with the need to collect information at a granular level to enable analysis of service use and morbidity patterns, and for decision-support in implementing the National Health Care Strategy.

Challenges remain that will be addressed in the pricing schedule’s future refinements. The first challenge is to gain insight from claims data for use in fraud and abuse prevention, clinical quality assessments, and cost-efficiency evaluations.

The second is to ensure a provider structure based on a sustainable payment model that allows providers to sustain their business model, and enables payers to avoid unsustainable cost increases over time. Qatar’s provider structure is diverse and further costing data will generate insights into the relationship between cost, quality, and prices. This will enable the introduction of models that reward providers for quality of care, while retaining the overall philosophy of a standard price list.

The final challenge is to leverage the specific opportunities provided by a new payment system to introduce innovative models of payments and incentives — especially with respect to bundled payments for outpatient services.

The steps taken thus far provide a beneficial starting point for making such improvements in the future.

